# Draft Genome Sequence of Plant Growth-Promoting Rhizobacterium *Burkholderia* sp. Strain USMB20, Isolated from Nodules of *Mucuna bracteata*

**DOI:** 10.1128/MRA.01051-20

**Published:** 2021-03-18

**Authors:** Nurohaida Ab Aziz, Salwani Shaffie, Ahmad Yamin Abdul Rahman, Yam Hokchai, Nazalan Najimudin, Amir Hamzah Ahmad Ghazali

**Affiliations:** a School of Biological Sciences, Universiti Sains Malaysia, Minden, Penang, Malaysia; b Faculty of Applied Science, UCSI University, Cheras, Kuala Lumpur, Malaysia; University of Southern California

## Abstract

*Burkholderia* sp. strain USMB20 is a plant growth-promoting rhizobacterium that was isolated from nodules of the leguminous cover crop *Mucuna bracteata.* The draft genome sequence of *Burkholderia* sp. strain USMB20 has an assembly size of 7.7 Mbp in 26 contigs with a GC content of 66.88%.

## Abstract (precis)

*Burkholderia* sp. strain USMB20 is a plant growth-promoting rhizobacterium that was isolated from nodules of the leguminous cover crop *Mucuna bracteata.* The draft genome sequence of *Burkholderia* sp. strain USMB20 has an assembly size of 7.7 Mbp in 26 contigs, with a GC content of 66.88%.

## ANNOUNCEMENT

Members of the genus *Burkholderia* occupy a wide range of ecological niches. Numerous strains are generally regarded as animal and human pathogens, while some are beneficial to plants (1–5). *Burkholderia* sp. strain USMB20 is a plant growth-promoting rhizobacterium that was isolated from the surface-sterilized nodules of a leguminous cover crop plant known as *Mucuna bracteata* (6, 7). This strain was deposited at the Microbial Culture Collection Unit, Institute of Bioscience, Universiti Putra Malaysia, with the accession number UPMC 404. The strain was grown at 28°C in yeast extract-mannitol agar (YEMA) medium containing bromothymol blue and was purified by restreaking twice (6). For DNA extraction, a single bacterial colony was cultured in lysogeny broth at 37°C for 16 h. The genomic DNA was extracted using the Qiagen genomic DNA extraction kit and purified using the Genomic-tip 100/G (Qiagen, Germany). The genome was sequenced using the Illumina and Pacific Biosciences (PacBio) sequencing technologies. An Illumina (CA, USA) library was prepared with the TruSeq DNA sample preparation kit. Illumina reads were obtained using a HiSeq 2000 platform at Macrogen, Inc. (South Korea), yielding 12,739,044    paired-end 100-bp reads. Long reads were obtained using PacBio RS single-molecule real-time (SMRT) technology with P4-C2 chemistry at DNA Link, Inc. (South Korea). A 10-kb library was prepared with the SMRTbell template preparation kit v1.0 (Pacific Biosciences, USA); it yielded 143,652,980 bases from 55,067 reads, with an average read length of 2,608 bp and an *N*_50_ value of 3,739 bp. Default parameters were used for all software unless otherwise specified. The paired-end short reads were filtered using the NGS QC Toolkit v2.3 (8) with a read length cutoff value of 70% and a base Phred quality score threshold of 20. The PacBio reads were subjected to Illumina-based correction by applying the long-read error correction tool in LSC software v0.3.1 (9). The cleaned Illumina and PacBio reads were *de novo* hybrid-assembled using the SPAdes assembler v3.12.0 (10) with 349.5-fold contig coverages. A customized Perl script with contig coverage of more than 5 and length above 200 bp as criteria was used for filtering small and low-coverage contigs (https://github.com/nurohaida/USMB20_project.git). The draft genome of *Burkholderia* sp. strain USMB20 was annotated using the NCBI Prokaryotic Genome Annotation Pipeline (PGAP) v4.8 (11).

The total size of the draft genome of *Burkholderia* sp. strain USMB20 was 7,700,664 bp, with an average GC content of 66.88%. The final assembly generated 26 contigs, with an *N*_50_ value of 696,911 bp. The genome contained 7,125 predicted coding sequences, of which 6,685 and 65 were identified as protein-coding and RNA-coding genes, respectively. The genome contained 139 genes related to motility and chemotaxis, which are important for plant colonization. *Burkholderia* sp. strain USMB20 possessed beneficial genes such as those for 1-aminocyclopropane-1-carboxylic acid (ACC) deaminase, indole-3-acetic acid (IAA) biosynthesis, siderophore and iron uptake, and phosphate solubilization for growth promotion of its host plant. Additionally, heavy metal (arsenic, copper, and chromium) resistance genes were identified in the sequenced genome.

### Data availability.

This whole-genome shotgun project was deposited in GenBank under the accession number JTAN00000000. The version described in this paper is the second version, JTAN02000000. Raw sequence reads were deposited in the GenBank Sequence Read Archive (SRA) under the accession numbers SRX9172553 and SRX9172554.
